# Identification of Key Functional Modules and Immunomodulatory Regulators of Hepatocellular Carcinoma

**DOI:** 10.1155/2021/1801873

**Published:** 2021-08-13

**Authors:** Ding Luo, Xiang Zhang, Xiao-Kai Li, Gang Chen

**Affiliations:** ^1^Department of Hepatobiliary Surgery, The First People's Hospital of Kunming City & Ganmei Affiliated Hospital of Kunming Medical University, No. 504 Youth Road, Kunming 650000, China; ^2^Department of Hepatobiliary Surgery, First Affiliated Hospital of Kunming Medical University, No. 295 Xichang Road, Kunming 650032, China

## Abstract

Despite the advances in the treatment of hepatocellular carcinoma (HCC), the prognosis of HCC patients remains unsatisfactory due to postsurgical recurrence and treatment resistance. Therefore, it is important to reveal the mechanisms underlying HCC and identify potential therapeutic targets against HCC, which could facilitate the development of novel therapies. Based on 12 HCC samples and 12 paired paracancerous normal tissues, we identified differentially expressed mRNAs and lncRNAs using the “limma” package in R software. Moreover, we used the weighted gene coexpression network analysis (WGCNA) to analyze the expression data and screened hub genes. Furthermore, we performed pathway enrichment analysis based on the Kyoto Encyclopedia of Genes and Genomes (KEGG) database. In addition, the relative abundance of a given gene set was estimated by single-sample Gene Set Enrichment Analysis. We identified 687 differentially expressed mRNAs and 260 differentially expressed lncRNAs. A total of 6 modules were revealed by WGCNA, and MT1M and MT1E genes from the red module were identified as hub genes. Moreover, pathway analysis revealed the top 10 enriched KEGG pathways of upregulated or downregulated genes. Additionally, we also found that CD58 might act as an immune checkpoint gene in HCC via PD1/CTLA4 pathways and regulate the levels of tumor-infiltrating immune cells in HCC tissues, which might be an immunotherapeutic target in HCC. Our research identified key functional modules and immunomodulatory regulators for HCC, which might offer novel diagnostic biomarkers and/or therapeutic targets for cancer immunotherapy.

## 1. Introduction

Hepatocellular carcinoma (HCC) is one of the most common malignant tumors and the fourth leading cause of cancer-related death globally, which has posed a substantial financial and health burden [1]. In China, HCC accounts for about 90% of all cases of primary liver cancer, and 422,100 individuals succumb to liver cancer annually [2]. Despite the advances in HCC treatment (especially immunotherapy), its mortality holds an increasing trend and the prognosis of patients with HCC remains unsatisfactory. However, the complex mechanisms underlying the initiation and progression of HCC make it challenging to develop novel therapeutic strategies.

Accumulating studies reveal the critical roles of immune cells in the initiation, metastasis, and recurrence of HCC [3, 4]. Multiple regulatory molecules could inhibit the antitumor activity of tumor-associated immune cells, thus resulting in immune escape [5–7]. As a strategy to normalize the antitumor immune responses against cancer cells, cancer immunotherapy has achieved clinical success in the last five years and revolutionized the treatment landscape of HCC [8]. However, the limited response and significant toxicity impede the therapeutic effect. Therefore, it is necessary to reveal the underlying mechanisms of HCC and identify potential therapeutic targets, with an emphasis on immunomodulatory regulators.

This study identified differentially expressed genes and lncRNAs based on the expression profile of 12 HCC samples and 12 paired paracancerous normal tissues. The weighted gene coexpression network analysis (WGCNA) was used to screen vital modules and hub genes correlated to HCC. Moreover, we performed pathway enrichment analysis based on the Kyoto Encyclopedia of Genes and Genomes (KEGG) database. In addition, we estimated the relative abundance of a given gene set by single-sample Gene Set Enrichment Analysis (ssGSEA). This study is aimed at identifying key functional modules and immunomodulatory regulators for HCC, which might offer novel diagnostic biomarkers and/or therapeutic targets for cancer immunotherapy.

## 2. Materials and Methods

### 2.1. Data Acquisition and Preprocessing

The expression profile of GSE115018 was downloaded from the Gene Expression Omnibus database. In GSE115018, Shi et al. [9] collected 12 HCC samples and 12 paired paracancerous normal tissues from the first affiliated hospital of Guangxi Medical University. All the patients received the first operation on primary disease without radiochemotherapy. The diagnosis of HCC was further confirmed using histopathology after surgery. Within 30 minutes after isolation, the tissues were immediately frozen in liquid nitrogen and stored at -80°C. The Ethics Committee of the First Affiliated Hospital of Guangxi Medical University approved this study, and informed consent for participation was acquired from all participants and their families [9]. We matched probes with gene symbols after removing redundant data (time, null value, etc.). A total of 9,945 mRNAs and 5,072 lncRNAs were analyzed.

### 2.2. Differential Expression Analysis

We first used the “wateRmelon” package to correct background, normalize quantile, and summarize quantile to eliminate potential error. Then, we used the “limma” package to analyze the differentially expressed mRNAs and lncRNAs between HCC and paired paracancerous normal tissues with criteria of adjusted *P* value < 0.05 and ∣log2 fold − change (FC) | ≥1.

### 2.3. WGCNA and Screening of Hub Genes

Weighted gene coexpression network analysis (WGCNA) is a systematic biology method [10], which identifies highly correlated genes and related modules to external sample traits. Before network construction, we removed obvious outlier samples or samples with excessive numbers of missing entries to avoid data deviation. Then, the step-by-step network construction and module detection were performed [10].

According to the criterion of approximate scale-free topology, we chose the soft-threshold power (*β*-value) of 7 to determine a scale-free topology index (*R*^2^) of 0.86. Then, we calculated adjacencies using the soft-threshold power and transformed the adjacency into the Topological Overlap Matrix (TOM), which was an average linkage hierarchical clustering with a dissimilarity measure to detect gene modules. The minimum module size of 30 and cut height of 0.99 were set to identify and merge gene modules with similar expression probes.

Furthermore, we used Zsummary to evaluate the functional module preservation [11]. Zsummary is comprised of four statistics related to density and three connectivity-related statistics, and it was created to quantitatively assess whether the density and connectivity patterns of modules defined in a reference dataset are preserved in a test dataset. A Zsummary value between 2 and 10 indicates moderate module preservation, whereas a Zsummary > 10 provides strong support for module preservation.

Genes with the highest degree of connectivity in a module were identified as hub genes, which could determine the biological significance of the module. We correlated the different module eigengenes (MEs) and the clinical traits. The gene significance (GS) quantifies the association of individual genes with the clinically interesting trait, and the module membership (MM) acts as the correlation between MEs and the gene expression profiles. Hub genes would be chosen if GS value was >-log10 (0.05) and the absolute value of kME was >0.85.

### 2.4. Pathway Enrichment Analysis

Kyoto Encyclopedia of Genes and Genomes (KEGG) is a database resource for understanding high-level functions and utilities of the biological system from molecular-level information, especially large-scale molecular datasets generated by genome sequencing and other high-throughput experimental technologies [12]. The logFC of DEGs obtained from differential expression analysis was applied for enrichment analysis. R packages of “clusterProfiler” and “enrichplot” were used to perform KEGG enrichment analysis with a threshold of *P* value <0.05. Further, enriched signaling pathways were visualized with “dotplot” and “gseaplot” packages of R software.

### 2.5. Network Analysis and Target Relationship Prediction

Coexpression network analysis was conducted based on the Pearson correlation coefficient to explore the correlations between differentially expressed lncRNAs and mRNAs. R was applied to the output node and edge files of the eligible paired lncRNA-mRNA interaction, and Cytoscape 3.6.0 software was used to visualize the lncRNA-mRNA coexpression network.

Then, miRNA targets of lncRNAs and miRNAs were carried out based on the miRanda database (http://www.microrna.org/) and TargetScan 7.2 (http://www.targetscan.org/vert_72/). The miRNAs cotargeting the certain lncRNA and the certain mRNA were filtered using Venny 2.1.0 (http://bioinfogp.cnb.csic.es/tools/venny/).

### 2.6. Expression Analysis of Hub Genes Based on TCGA

The expression of hub genes in HCC and normal tissues was evaluated using the UALCAN, an interactive web portal to perform in-depth analyses of TCGA gene expression data [13]. Meanwhile, the expression levels of hub genes were also evaluated in patients with various tumor grades, ages, genders, and races.

### 2.7. TIMER Database Analysis

We used the TIMER database to analyze the association between the hub gene expression and the abundance of infiltrating immune cells, including B cells, CD8^+^ T cells, CD4^+^ T cells, macrophages, neutrophils, and dendritic cells [14].

### 2.8. The Immune Cell Infiltration in Tumor Cells

We comprehensively estimated the infiltration level of immune cell populations of each sample by ssGSEA, which was implemented in the GSVA package [15]. As an extended gene set enrichment analysis method, ssGSEA was designed to compute the separate enrichment scores for a particular gene set in each sample instead of the gene-phenotype association score. The gene set from a previous study [16], which included 364 genes representing 24 microenvironment cell types (see their Supplementary Table S1), was input into the ssGSEA algorithm. The overall immune cell infiltrating levels were estimated by the R estimate package [17].

## 3. Result

### 3.1. DEG Identification

**Figure 1:**
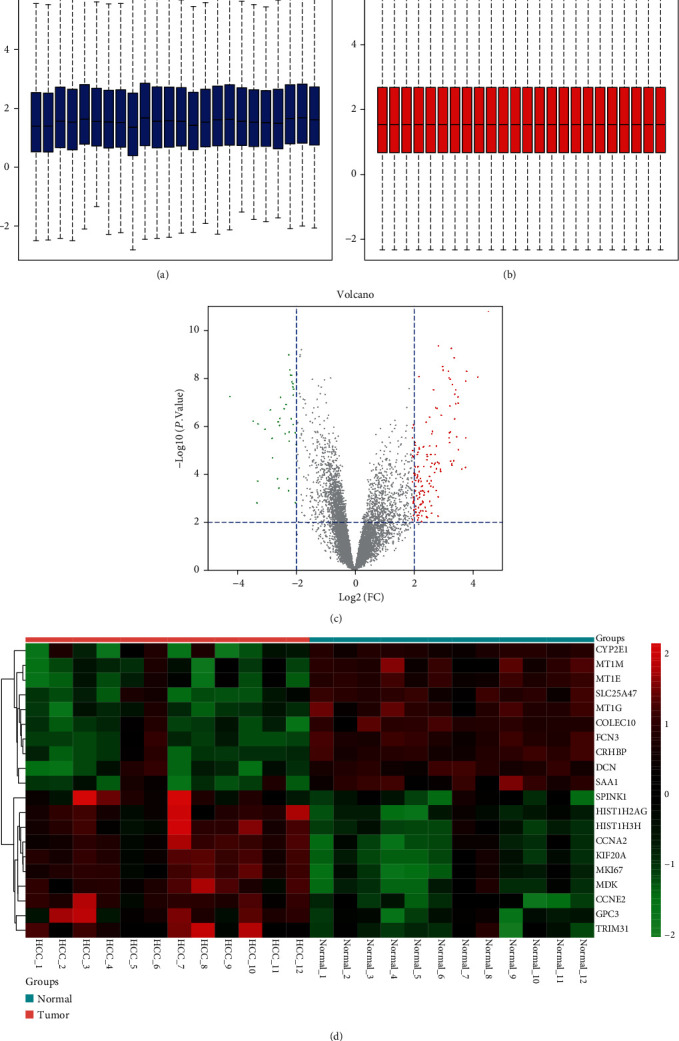
Identification of differentially expressed mRNAs. Box plots show the distribution of the relative mRNA expression in each sample before (a) and after (b) normalization of GSE115018. Each box corresponds to one sample. The middle line corresponds to the median. (c) Volcano plot of differentially expressed mRNAs. Differentially expressed mRNAs were screened with criteria of adjusted *P* value < 0.05 and ∣log2 fold − change (FC) | ≥1. (d) The cluster heat map of the top 10 upregulated and downregulated mRNAs.

After correcting background, normalizing quantile, and summarizing quantile (Figures 1(a) and 1(b)), we identified a total of 687 differentially expressed mRNAs in HCC tissues compared with paracancerous normal tissues.  Figure 1(c) shows the volcano plot of differentially expressed mRNAs. The top 10 differentially upregulated genes (including *SPINK1*, *HIST1H2AG*, *HIST1H3H*, *CCNA2*, *KIF20A*, *MKI67*, *MDK*, *CCNE2*, *GPC3*, and *TRIM31*) and top 10 downregulated genes (including *CYP2E1*, *MT1M*, *MT1E*, *SLC25A47*, *MT1G*, *COLEC10*, *FCN3*, *CRHBP*, *DCN*, and *SAA1*) are shown in the heat map (Figure 1(d)).

**Figure 2:**
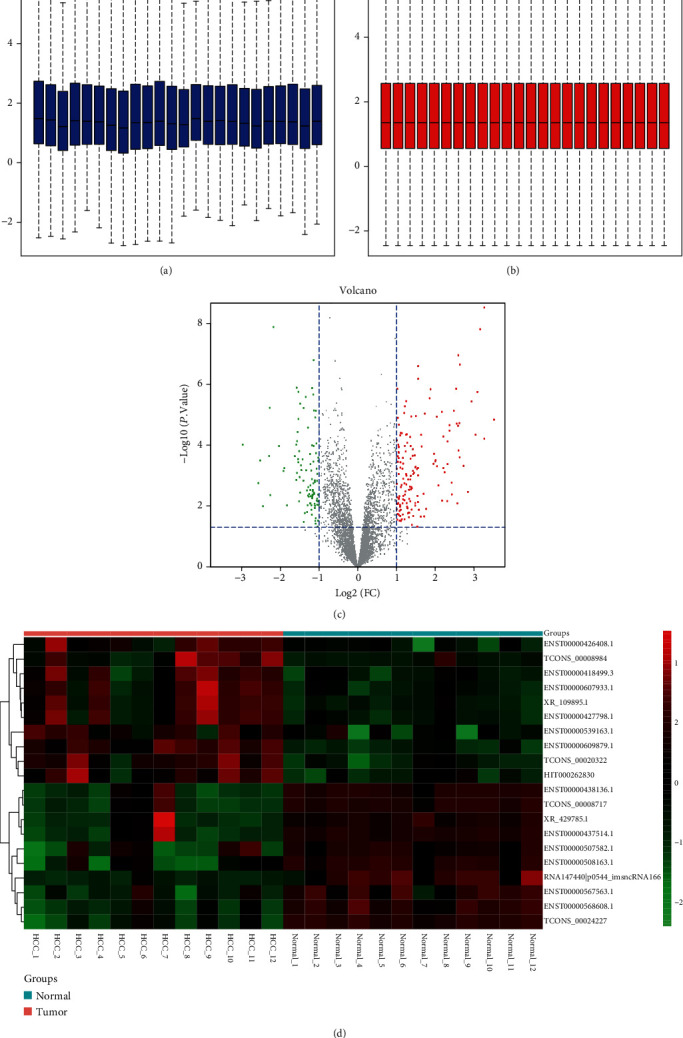
Identification of differentially expressed lncRNAs. Box plots show the distribution of the relative lncRNA expression in each sample before (a) and after (b) normalization of GSE115018. (c) Volcano plot of differentially expressed lncRNAs. Differentially expressed lncRNAs were screened with criteria of adjusted *P* value < 0.05 and ∣log2 fold − change (FC) | ≥1. (d) The cluster heat map of the top 10 upregulated and downregulated lncRNAs.

Moreover, a total of 260 differentially expressed lncRNAs were identified from dataset GSE115018 after preprocessing (Figures 2(a) and 2(b)). As shown in the volcano plot (Figure 2(c)), 102 of these lncRNAs were upregulated in the tumor, while 158 of them were downregulated. The top 10 upregulated and downregulated lncRNAs in HCC tissues compared with paracancerous normal tissues are shown in the heat map (Figure 2(d)).

### 3.2. WGCNA and Screening of Hub Genes

**Figure 3:**
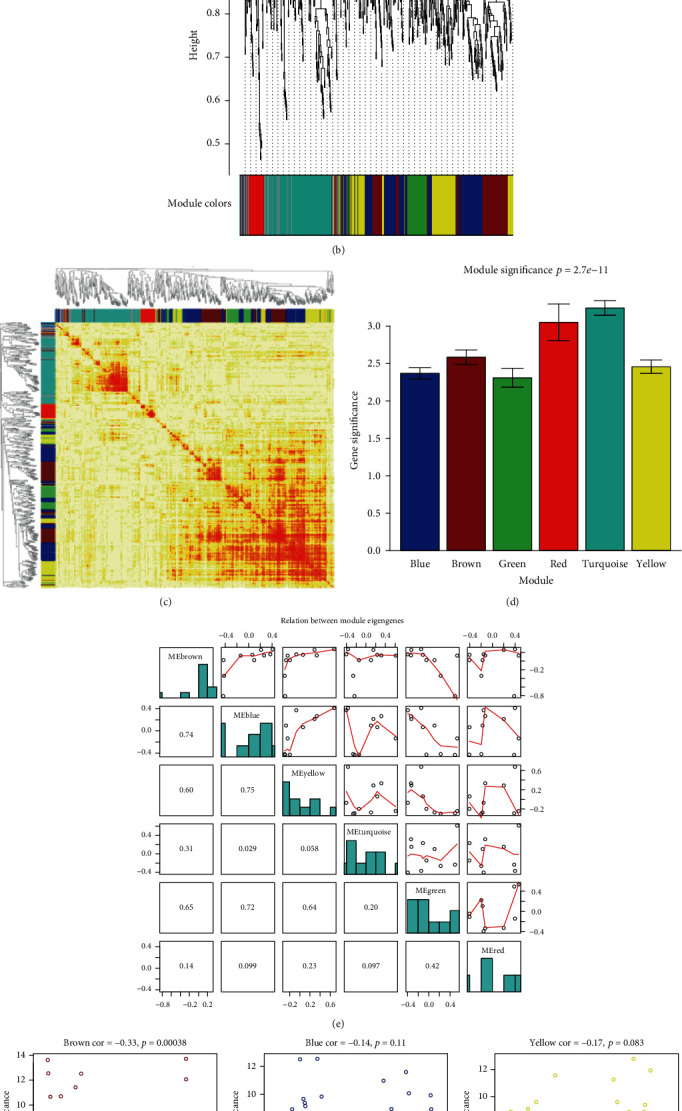
Construction of weighted coexpression network and module analysis. (a) Soft-threshold selection. A soft-threshold power of 7 with an *R*^2^ of 0.87 was selected. (b) Cluster dendrogram. Each color indicates one specific coexpression module, and each vertical line indicated an individual gene. Six modules were identified in WGCNA, including blue, brown, green, red, turquoise, and yellow module. (c) Network heat map plot of all genes suggesting the interaction of coexpression genes via TOM dissimilarity. The axe colors indicate the respective modules. The color intensity represents the degree of overlap, where a darker yellow suggests a higher degree of connectivity. (d) Barplot of mean gene significance associated with HCC. A higher value shows a more significant relationship. (e) Relation between different module eigengenes. (f) Scatterplots of genes in the six modules. Module membership is set as the *x*-axis, whereas the *y*-axis is gene significance.

To identify key modules related to HCC, the soft-threshold power of 7 was set to ensure a scale-free network (scale *R*^2^ = 0.86) (Figure 3(a)). With a minModuleSize of 30 and a CutHeight of 0.99, a total of six modules were identified in WGCNA: blue, brown, green, red, turquoise, and yellow module (Figure 3(b)). The heat map depicting the TOM of genes is shown in Figure 3(c). The darker parts indicated a higher degree of connectivity and suggested that these genes might be highly related to HCC. The results of GS showed that the module significance (MS) of the turquoise and red modules was higher than that of the other modules. The module eigengene tree showed a similarity among different modules (Figure 3(d)). The module yellow and module blue had high similarity, and the correlation coefficient between these two modules was 0.75. Scatterplots of module membership and GS showed where each gene was in the relevant network (Figures 3(e) and 3(f)). Additionally, we also performed enrichment analysis on the blue and yellow modules using Gene Ontology (GO) and the KEGG database. As shown in SFigure 1A, the enriched biological processes were mainly involved in fatty acid metabolic process, response to xenobiotic stimulus, cellular response to xenobiotic stimulus, xenobiotic metabolic process, and drug catabolic process. The cellular components were primarily enriched in mitochondrial matrix and mitochondrial inner membrane, whereas the enriched molecular functions were mainly associated with steroid hydroxylase activity and monooxygenase activity. KEGG pathway analysis suggested that the metabolism of xenobiotics by cytochrome P450 was the most enriched pathway, followed by complement and coagulation cascades, glucagon signaling pathway, lipid and atherosclerosis, and PI3K-Akt signaling pathway (SFigure 1B).

**Figure 4:**
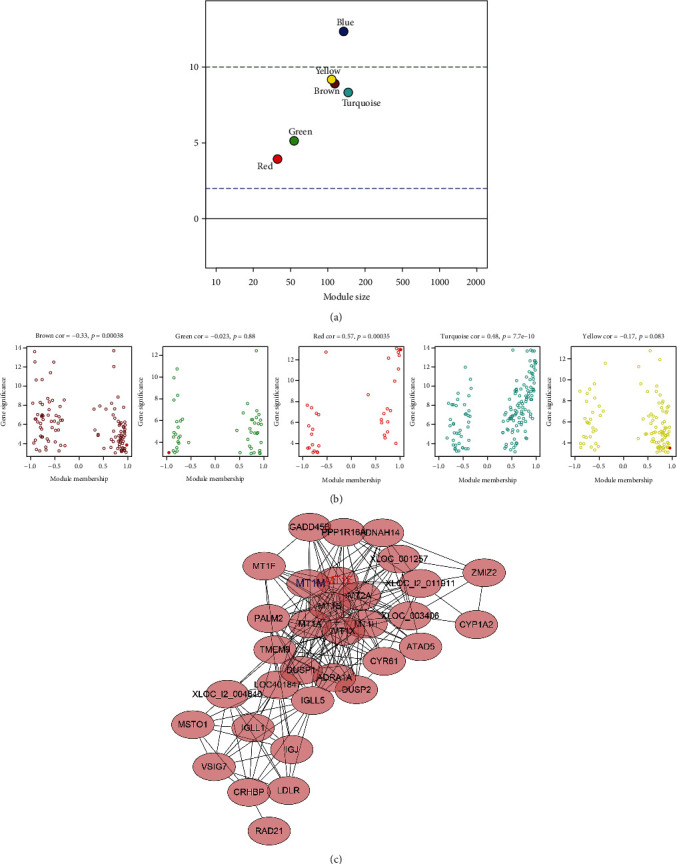
The Zsummary statistics of the module preservation and hub gene screening. (a) Zsummary score analysis of different modules. The dashed blue and green lines suggested the thresholds of 2 and 10, respectively. A Zsummary value between 2 and 10 indicates moderate module preservation, whereas a Zsummary > 10 provides strong support for module preservation. (b) Hub genes in different modules. The red solid points present the hub genes in each module. (c) Network of genes in the red module.

Zsummary of brown, green, red, turquoise, and yellow modules were all <10 (Figure 4(a)), and we chose these five modules to perform further analysis and identify hub genes. With a threshold of GS > −log10 (0.05) and the absolute value of kME > 0.85, we selected hub genes and intersected with the top 10 upregulated or downregulated ones. Hub genes in the five modules are shown in Figure 4(b). Importantly, *MT1M* and *MT1E* genes from the red module were identified as hub genes. Moreover, we constructed a network for genes from the red module, and *MT1E* and *MT1M* genes were in the network center (Figure 4(c)).

### 3.3. KEGG Pathway Analysis

**Figure 5:**
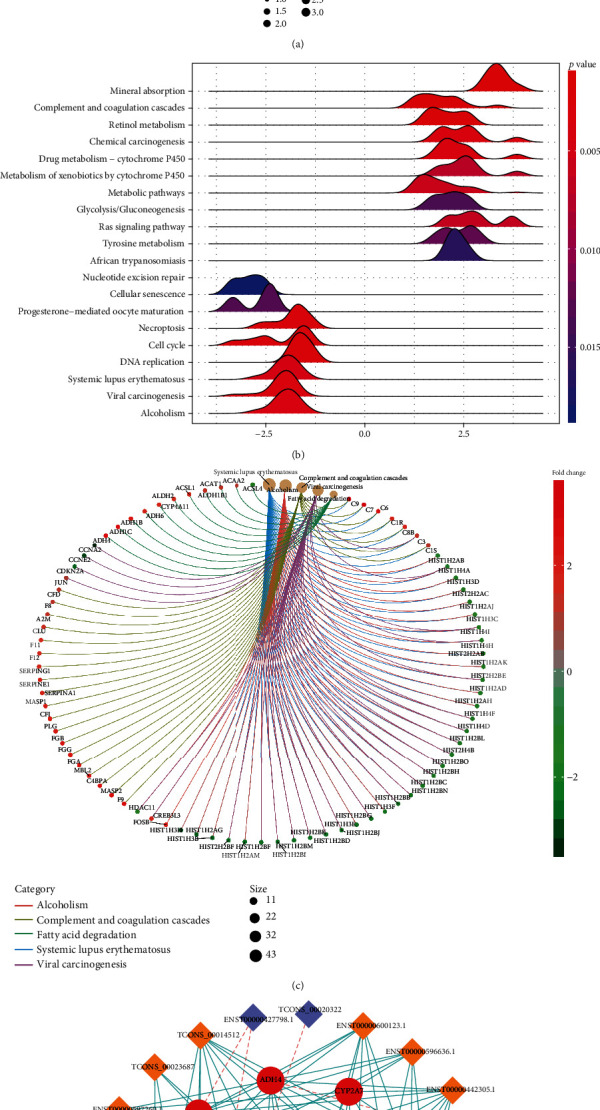
KEGG pathway analysis and coexpression network. (a) Dot plot of the top 10 enriched KEGG pathways of upregulated or downregulated genes. The logFC of DEGs obtained from differential expression analysis was applied for enrichment analysis. (b) The mountain range of the top 10 enriched KEGG pathways of upregulated or downregulated genes. (c) Cnetplot of the correlation between metabolism pathways and differentially expressed mRNAs. (d) Coexpression of lncRNA and mRNA network from the retinol metabolism pathway.

We performed KEGG pathway analysis to achieve a more in-depth insight into the biological roles of the identified DEGs. Figures 5(a) and 5(b) show the top 10 enriched KEGG pathways of upregulated and downregulated genes. For the upregulated genes, complement and coagulation cascades, retinol metabolism, and glycolysis were the top three pathways. Moreover, DNA replication, alcoholism, and viral carcinogenesis were the top three enriched pathways for downregulated genes. Different genes enriched by these pathways were shown in Figure 5(c). Notably, the retinol metabolism pathway was highly expressed in HCC, and the coexpression network of lncRNAs and mRNAs from the retinol metabolism pathway was shown in Figure 5(d).

### 3.4. Expression Analysis of MT1E and MT1M Based on TCGA

**Figure 6:**
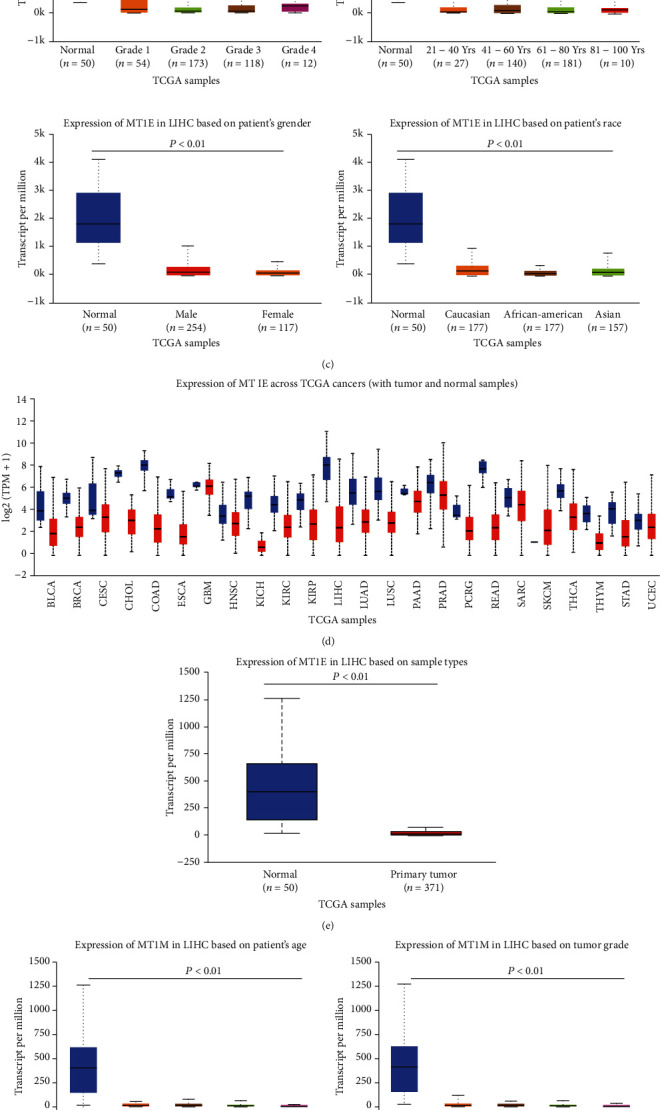
*MT1E* and *MT1M* expression levels in HCC based on UALCAN portal analysis and their association with immune cell infiltration. (a) Boxplot showing the overall comparison of *MT1E* expression between tumor and normal samples across multiple cancer types in the TCGA database. (b) The expression of *MT1E* in normal tissues compared with HCC tissues. (c) The comparison of *MT1E* expression in normal tissues compared with HCC tissues from different tumor grade (grades 1 to 4), age (21-40, 41-60, 61-80, and 81-100 years old), gender (male and female), and race (Caucasian, African-American, or Asian ethnicity). (d) Boxplot showing the overall comparison of *MT1M* expression between tumor and normal samples across multiple cancer types in the TCGA database. (e) The expression of *MT1M* in normal tissues compared with HCC tissues. (f) The comparison of *MT1M* expression in normal tissues compared with HCC tissues from different tumor grade (grades 1 to 4), age (21-40, 41-60, 61-80, and 81-100 years old), gender (male and female), and race (Caucasian, African-American, or Asian ethnicity). (g) Correlation of *MT1E* and *MT1M* expression with immune infiltration level in HCC via the TIMER database. Both *MT1E* and *MT1M* expression were significantly correlated with the infiltration levels of B cells, CD8^+^ T cells, CD4^+^ T cells, macrophages, neutrophils, and dendritic cells in HCC.

Figures 6(a) and 6(d) show an overview on the *MT1E* and *MT1M* expression levels in tumors compared with normal samples across multiple cancer types in the TCGA database. *MT1E* and *MT1M* were significantly downregulated in HCC tissues than in normal samples (both *P* value < 0.01; Figures 6(b) and 6(e)). Moreover, Figures 6(c) and 6(f) showed that this trend was consistent regardless of tumor grade (grades 1 to 4), age (21-40, 41-60, 61-80, and 81-100 years old), gender (male and female), and race (Caucasian, African-American, or Asian ethnicity).

### 3.5. TIMER Database Analysis

We used the TIMER database to explore the correlation between the expression level of *MT1E* and *MT1M* and immune cell infiltration. Both MT1E and MT1M expression were significantly correlated with the infiltration levels of B cells, CD8^+^ T cells, CD4^+^ T cells, macrophages, neutrophils, and dendritic cells in HCC. Furthermore, there was a negative correlation between *MT1E* expression and the infiltration of B cells, CD4^+^ T cells, and macrophages, while the expression of *MT1M* was positively associated with the infiltration of B cells, CD8^+^ T cells, macrophages, neutrophils, and dendritic cells. These results indicated that *MT1E* and *MT1M* might play an important role in regulating immune infiltration in HCC (Figure 6(g).

### 3.6. The Association between Functional Modules and the Immunomodulatory Regulators in HCC

**Figure 7:**
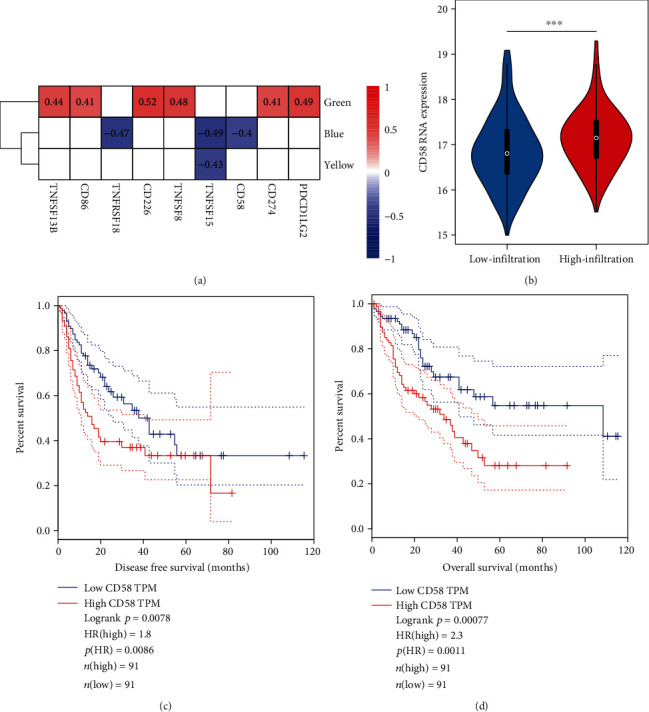
The association between functional modules and the immunomodulatory regulators in HCC. (a) The relative expression abundances of the functional modules estimated by ssGSEA. (b) The CD58 RNA expression level in tumor tissues. *CD58* was highly expressed in HCC tissues with high infiltrating levels of immune cells (*P* value < 0.001) (c) The disease-free survival and (d) the overall survival for patients with low (below median CD58 expression value, *n* = 91) or high (above median CD58 expression value, *n* = 91) expression level. Kaplan-Meier survival curves were generated based on TCGA data using the GEPIA2 tool.

To further explore the biological roles of the functional modules, we conducted a correlation analysis between the functional modules and immunomodulatory regulators in the TCGA liver cancer cohort, which had a larger sample size. The relative expression abundances of the functional modules were estimated by ssGSEA (Figure 7(a)). Specifically, we observed that the green module was positively correlated with *TNFSF13B*, *CD86*, *CD226*, *TNFSF8*, *CD274*, and *PDCD1LG2*. Particularly, *CD86*, *CD274*, and *PDCD1LG2* were well-recognized immune checkpoint genes involved in PD1/CTLA4 pathways. In contrast, the blue module was negatively correlated with *TNFRSF18* (correlation coefficient = −0.47), *TNFSF15* (correlation coefficient = −0.49), or *CD58* (correlation coefficient = −0.4), whereas yellow was negatively correlated with *TNFSF15* with a correlation coefficient of -0.43 (all *P* value < 0.05).

Among these immunomodulatory regulators, *CD58* was highly expressed in HCC tissues with high infiltrating levels of immune cells (Figure 7(b), Wilcoxon-rank sum test, *P* < 0.001). Furthermore, high *CD58* expression was associated with shorter disease-free survival (*P* value = 0.0086) and overall survival (*P* value = 0.0078), suggesting that *CD58* was closely associated with HCC prognosis (Figures 7(c) and 7(d)).

## 4. Discussion

This study identified 687 differentially expressed mRNAs and 260 differentially expressed lncRNAs based on 12 HCC samples and 12 paired paracancerous normal tissues. WGCNA revealed a total of 6 modules associated with HCC, and *MT1M* and *MT1E* from the red module were identified as hub genes. Then, the pathway enrichment analysis revealed the top 10 enriched KEGG pathways, and we created a coexpression network of lncRNAs and mRNAs from the retinol metabolism pathway. Interestingly, our research indicated that *CD58* might act as an immune checkpoint gene in HCC and regulate the levels of tumor-infiltrating immune cells in HCC tissues, which might be an immunotherapeutic target in HCC.

Despite the remarkable advances in the treatment strategies [18], HCC is still the fourth leading cause of cancer-related death globally, and it has posed a substantial health burden [19]. Bioinformatics technology provides a novel method to reveal the mechanisms underlying HCC and identify potential therapeutic targets, which contributes to the development of novel agents against HCC.

Metallothionein (MT) family members are reported to be associated with the cellular metabolism of metal ions and cancer development. For example, MT family members were reported to take part in the malignant transformation of hepatocytes [20]. They could also protect cells from anticancer agents and irradiation-induced damage [21]. *MT1* is one of the MT family members, which is expressed in all eukaryotes. It was reported that *MT1* was downregulated in HCC, and the silence of *MT1* could promote the proliferation of liver cancer [22]. Moreover, *MT1M* was demonstrated as a tumor suppressor gene downregulated in HCC, which would contribute to liver tumorigenesis by increasing cellular NF-*κ*B activity [23, 24]. Consistent with previous researches, our analysis discovered that *MT1M* and *MT1E* were downregulated in HCC tissue and identified them as hub genes in HCC. However, the role of *MT1E* in liver cancer is seldom studied. Our analysis disclosed that *MT1E* was downregulated in liver cancer and may serve a similar function as *MT1M*.

KEGG pathway analysis suggested that multiple pathways (including complement and coagulation cascades, retinol metabolism, glycolysis pathways, and so forth) were significantly enriched. Among them, the complement system and coagulation cascade are classic immunomodulatory regulators for both innate and adaptive immune responses [25]. Moreover, retinol (vitamin A) lies at metabolic crossroads of multiple biochemical reactions, which serve as a necessary precursor for retinoid and retinoic acid [26]. Immunomodulatory effects of retinoids have been revealed in various human cellular lineages, including thymocyte, lung fibroblast, Langerhans' cell, natural killer cell, peripheral blood mononuclear cell, and tumoral cell [27]. For example, retinoic acid significantly improves the expansion of Foxp3^+^ inducible regulatory T cell and inhibits the differentiation of T helper 17 cell, which contributes to maintaining immune homeostasis [28, 29]. In contrast, retinol deficiency increases proinflammatory cytokines and elevates T helper type 1 response [30]. The immunomodulatory roles of retinoids have been summarized in several reviews [31, 32]. The close connection between retinoids and liver diseases has also long been recognized [33, 34], and several experimental studies indicated the beneficial effects of retinoids on blocking HCC development [35–37]. Additionally, accumulating evidence suggests the immunomodulatory role of butyrate, which could affect the epigenetic status of immune cells via downregulating the enzymatic function of histone deacetylase [38–40].

In further network analysis on the retinoid metabolism pathway, *CYP1A2*, *CYP2A13*, *CYP2A7*, *CYP2B6*, *CYP2C19*, *CYP4A11*, *ADH1B*, *ADH1C*, *ADH4*, *ADH6*, and *RDH5* were in the center of the network. High expression of *CYP1A2* was reported to serve as a biomarker to predict recurrence-free survival of HCC [41]. Moreover, the *CYP2A13*, *CYP2A7*, *CYP2B6*, and *CYP2C19* genes belong to the CYP2 family [42]. *CYP2A13* is a human cytochrome P450 (P450) enzyme, which is widely expressed in the liver [43, 44]. Moreover, it is responsible for the metabolism of nicotine, coumarin, and tobacco-specific nitrosamine [45]. Recently, the interaction between *CYP2A13* and *ABCB1*was reported to be closely associated with lung cancer, and *CYP2A13* was identified as a potential critical metabolic enzyme gene in the carcinogenesis of lung cancer [46, 47]. Additionally, the *CYP2A7* pseudogene transcript was demonstrated to affect the expression of *CYP2A6* in the liver as a decoy for miR-126, but the role of *CYP2A7* in HCC remains vague [48]. *CYP2B6* and *CYP2C19* were previously reported as unfavorable prognosis markers in breast cancer [49, 50]. In the analysis of circulating DNA from patients with advanced hepatocellular carcinoma, *CYP2B6*, *BAX*, and *HNF1A* genes showed the highest mutation frequency and a significant association with the clinicopathological characteristics of HCC, which suggested potential roles as driver genes in a specific clinical setting [51]. *CYP2C19* belongs to cytochrome P2C subfamily members, which are known to be involved in clinical drug metabolism. The expression levels of *CYP2C8*, *CYP2C9*, and *CYP2C19* genes were identified as potential prognostic markers of HCC following hepatectomy [42]. Also, the downregulation of the *CYP2C19* gene is associated with aggressive tumor potential and poorer recurrence-free survival of HCC [52, 53]. In our analysis, *CYP2B6* and *CYP2C19* were discovered to be upregulated in HCC, and the roles in the process of liver cancer are required to be further verified.

*ADH4* is an important member of the ADH family, which was involved in metabolizing a large variety of substrates such as ethanol and retinol. ADH4 mRNA and protein expression levels were markedly reduced in HCC tissues and were reported to be recognized as potential prognostic biomarkers for HCC patients. HCC patients with lower ADH4 showed a worse overall survival rate compared with those with high expression (*P* < 0.001), and the expression of *ADH4* was an independent predictor of overall survival (HR, 0.154; 95% CI, 0.044-0.543; *P* = 0.004).

To further explore the biological roles of the functional modules, we also conducted a correlation analysis between the functional modules and immunomodulatory regulators. We found that green, blue, and yellow modules were closely associated with some immune checkpoint genes involved in PD1/CTLA4 pathways. The immune adhesion molecule *CD58*, also termed lymphocyte function-associated antigen-3 (LFA-3), is a costimulatory receptor extensively expressed on human cells [54, 55]. The interaction between *CD58* and its natural ligand (CD2) promotes optimal T/nature killer cell activation and triggers a series of intracellular signaling. Accumulating evidence has demonstrated the central role of CD2-CD58 interaction in modulating antiviral responses, inflammatory responses, and immune evasion of solid cancer cells [55, 56]. In gastric cancer, elevated expression of *CD58* was associated with deteriorated tumor cell invasion, reduced survival time, and cancer recurrence [57]. Consistently, our study showed that *CD58* might be an unfavorable prognostic gene, a higher expression of which was significantly correlated with shorter disease-free and overall survival. We found that *CD58* was highly expressed in tumor tissues with high infiltrating levels of immune cells, suggesting that the activated infiltrating immune cells in tumor tissues might also be suppressed by *CD58*. Interestingly, *CD58* loss was observed to induce immune evasion in multiple melanoma cell and tumor-infiltrating lymphocyte coculture models, and the expression of *CD58* was downregulated in melanoma patients receiving immune checkpoint inhibitors [56]. Also, PD-L1 was upregulated in *CD58*-knockout melanoma cells. Their results suggested that *CD58* downregulation (or loss) might contribute to immune evasion via multiple distinct mechanisms (e.g., increased expression of coinhibitory PD-L1) [56]. Additional studies are necessary to investigate the role of *CD58* in HCC and *CD58*-PD-L1 balance.

Furthermore, large amounts of lncRNAs have been demonstrated to be abnormally expressed in HCC, and they play essential roles in cancer development, proliferation, and differentiation [58–60]. LncRNAs could regulate the expression of mRNA in various ways both in *cis*, and *trans* [61], and lncRNA can also act as sponges of mRNA or work with protein to regulate the expression of mRNA [62, 63]. Our results shed light on the interaction of mRNA and lncRNA and indicated the important roles of lncRNAs in HCC. Still, the lack of confirmatory experiments is a significant limitation, and more studies are required to validate the results further.

## 5. Conclusion

Our research identified key functional modules and immunomodulatory regulators for HCC, which might offer novel diagnostic biomarkers and/or therapeutic targets for cancer immunotherapy.

## Data Availability

The expression profile of GSE115018 was downloaded from Gene Expression Omnibus (GEO) database (http://www.ncbi.nlm.nih.gov/geo/).

## Supplementary Materials

Supplementary Materials SFigure 1: enrichment analysis on the blue and yellow modules using (A) Gene Ontology and the (B) KEGG database. The significance of enrichment gradually increases from blue to red, and the dot size indicates the number of genes contained in the corresponding enriched term.

## References

[1] Bray F., Ferlay J., Soerjomataram I., Siegel R. L., Torre L. A., Jemal A. (2018). Global cancer statistics 2018: Globocan estimates of incidence and mortality worldwide for 36 cancers in 185 countries. *CA: A Cancer Journal for Clinicians*.

[2] Chen W., Zheng R., Baade P. D. (2016). Cancer statistics in China, 2015. *CA: A Cancer Journal for Clinicians*.

[3] Eggert T., Wolter K., Ji J. (2016). Distinct functions of senescence-associated immune responses in liver tumor surveillance and tumor progression. *Cancer Cell*.

[4] Zhang P. F., Wang F., Wu J. (2019). Lncrna SNHG3 induces EMT and sorafenib resistance by modulating the miR-128/CD151 pathway in hepatocellular carcinoma. *Journal of Cellular Physiology*.

[5] Chen J., Gingold J. A., Su X. (2019). Immunomodulatory TGF-*β* signaling in hepatocellular carcinoma. *Trends in Molecular Medicine*.

[6] Liu L. Z., Zhang Z., Zheng B. H. (2019). CCL15 recruits suppressive monocytes to facilitate immune escape and disease progression in hepatocellular carcinoma. *Hepatology*.

[7] McGranahan N., Rosenthal R., Hiley C. T. (2017). Allele-specific HLA loss and immune escape in lung cancer evolution. *Cell*.

[8] Xu W., Liu K., Chen M. (2019). Immunotherapy for hepatocellular carcinoma: recent advances and future perspectives. *Therapeutic Advances in Medical Oncology*.

[9] Shi J., Ye G., Zhao G. (2018). Coordinative control of G2/M phase of the cell cycle by non-coding rnas in hepatocellular carcinoma. *PeerJ*.

[10] Langfelder P., Horvath S. (2008). WGCNA: an R package for weighted correlation network analysis. *BMC bioinformatics*.

[11] Heywang S. H., Fenzl G., Beck R. (1986). Anwendung von Gd-DTPA bei der kernspintomographischen Untersuchung der Mamma. *RoFo : Fortschritte auf dem Gebiete der Rontgenstrahlen und der Nuklearmedizin*.

[12] Kanehisa M., Goto S., Sato Y., Furumichi M., Tanabe M. (2012). KEGG for integration and interpretation of large-scale molecular data sets. *Nucleic Acids Research*.

[13] Chandrashekar D. S., Bashel B., Balasubramanya S. A. H. (2017). UALCAN: a portal for facilitating tumor subgroup gene expression and survival analyses. *Neoplasia*.

[14] Li T., Fan J., Wang B. (2017). Timer: a web server for comprehensive analysis of tumor-infiltrating immune cells. *Cancer Research*.

[15] Hanzelmann S., Castelo R., Guinney J. (2013). GSVA: gene set variation analysis for microarray and RNA-seq data. *BMC bioinformatics*.

[16] Xiao Y., Ma D., Zhao S. (2019). Multi-omics profiling reveals distinct microenvironment characterization and suggests immune escape mechanisms of triple-negative breast cancer. *Clinical Cancer Research*.

[17] Yoshihara K., Shahmoradgoli M., Martinez E. (2013). Inferring tumour purity and stromal and immune cell admixture from expression data. *Nature Communications*.

[18] Sun J. Y., Yin T. L., Zhou J., Xu J., Lu X. J. (2020). Gut microbiome and cancer immunotherapy. *Journal of Cellular Physiology*.

[19] El-Serag H. B., Rudolph K. L. (2007). Hepatocellular carcinoma: epidemiology and molecular carcinogenesis. *Gastroenterology*.

[20] Tanimoto K., Akbar S. M., Yamauchi Y., Michitaka K., Horiike N., Onji M. (1998). Immunohistochemical localization of metallothionein in hepatocellular carcinoma: preferential expression in non-cancerous cirrhotic nodules. *Oncology Reports*.

[21] Theocharis S. E., Margeli A. P., Klijanienko J. T., Kouraklis G. P. (2004). Metallothionein expression in human neoplasia. *Histopathology*.

[22] Datta J., Majumder S., Kutay H. (2007). Metallothionein expression is suppressed in primary human hepatocellular carcinomas and is mediated through inactivation of ccaat/enhancer binding protein alpha by phosphatidylinositol 3-kinase signaling cascade. *Cancer Research*.

[23] Ji X. F., Fan Y. C., Gao S., Yang Y., Zhang J. J., Wang K. (2014). MT1M and MT1G promoter methylation as biomarkers for hepatocellular carcinoma. *World Journal of Gastroenterology*.

[24] Mao J., Yu H., Wang C. (2012). Metallothionein MT1M is a tumor suppressor of human hepatocellular carcinomas. *Carcinogenesis*.

[25] Dunkelberger J. R., Song W. C. (2010). Complement and its role in innate and adaptive immune responses. *Cell Research*.

[26] D’Ambrosio D. N., Clugston R. D., Blaner W. S. (2011). Vitamin A metabolism: an update. *Nutrients*.

[27] Allende L. M., Corell A., Madrono A. (1997). Retinol (vitamin A) is a cofactor in CD3-induced human T-lymphocyte activation. *Immunology*.

[28] Mucida D., Park Y., Cheroutre H. (2009). From the diet to the nucleus: vitamin A and TGF-*β* join efforts at the mucosal interface of the intestine. *Seminars in Immunology*.

[29] Schuhmann M. K., Stegner D., Berna-Erro A. (2010). Stromal interaction molecules 1 and 2 are key regulators of autoreactive T cell activation in murine autoimmune central nervous system inflammation. *The Journal of Immunology*.

[30] Garcia O. P. (2012). Effect of vitamin a deficiency on the immune response in obesity. *Proceedings of the Nutrition Society*.

[31] Oliveira L. M., Teixeira F. M. E., Sato M. N. (2018). Impact of retinoic acid on immune cells and inflammatory diseases. *Mediators of Inflammation*.

[32] Patel S., Vajdy M. (2015). Induction of cellular and molecular immunomodulatory pathways by vitamin A and flavonoids. *Expert Opinion on Biological Therapy*.

[33] Cortes E., Lachowski D., Rice A. (2019). Retinoic acid receptor-beta is downregulated in hepatocellular carcinoma and cirrhosis and its expression inhibits myosin-driven activation and durotaxis in hepatic stellate cells. *Hepatology*.

[34] Shirakami Y., Sakai H., Shimizu M. (2015). Retinoid roles in blocking hepatocellular carcinoma. *Hepatobiliary Surgery and Nutrition*.

[35] Muto Y., Moriwaki H. (1984). Antitumor activity of vitamin A and its derivatives. *JNCI: Journal of the National Cancer Institute*.

[36] Shao R. X., Otsuka M., Kato N. (2005). Acyclic retinoid inhibits human hepatoma cell growth by suppressing fibroblast growth factor-mediated signaling pathways. *Gastroenterology*.

[37] Shimizu M., Suzui M., Deguchi A., Lim J. T., Weinstein I. B. (2004). Effects of acyclic retinoid on growth, cell cycle control, epidermal growth factor receptor signaling, and gene expression in human squamous cell carcinoma cells. *Clinical Cancer Research*.

[38] Arpaia N., Campbell C., Fan X., Dikiy S., van der Veeken J. (2013). Metabolites produced by commensal bacteria promote peripheral regulatory T-cell generation. *Nature*.

[39] Chang P. V., Hao L., Offermanns S., Medzhitov R. (2014). The microbial metabolite butyrate regulates intestinal macrophage function via histone deacetylase inhibition. *Proceedings of the National Academy of Sciences*.

[40] Mathewson N. D., Jenq R., Mathew A. V. (2016). Gut microbiome-derived metabolites modulate intestinal epithelial cell damage and mitigate graft-versus-host disease. *Nature Immunology*.

[41] Sciarra A., Pintea B., Nahm J. H. (2017). CYP1A2 is a predictor of HCC recurrence in HCV-related chronic liver disease: a retrospective multicentric validation study. *Digestive and Liver Disease: Official Journal of the Italian Society of Gastroenterology and the Italian Association for the Study of the Liver*.

[42] Wang X., Yu T., Liao X. (2018). The prognostic value of CYP2C subfamily genes in hepatocellular carcinoma. *Cancer Medicine*.

[43] Liu Z., Megaraj V., Li L., Sell S., Hu J., Ding X. (2015). Suppression of pulmonary CYP2A13 expression by carcinogen-induced lung tumorigenesis in a CYP2A13-humanized mouse model. *Drug Metabolism and Disposition*.

[44] Su T., Bao Z., Zhang Q. Y., Smith T. J., Hong J. Y., Ding X. (2000). Human cytochrome p450 CYP2A13: predominant expression in the respiratory tract and its high efficiency metabolic activation of a tobacco-specific carcinogen, 4-(methylnitrosamino)-1-(3-pyridyl)-1-butanone. *Cancer Research*.

[45] Kumondai M., Hosono H., Maekawa M. (2018). Functional characterization of 9 CYP2A13 allelic variants by assessment of nicotine C-oxidation and coumarin 7-hydroxylation. *Drug Metabolism and Pharmacokinetics*.

[46] Hua F., Guo Y., Sun Q., Yang L., Gao F. (2019). Hapmap-based study: cyp2a13 may be a potential key metabolic enzyme gene in the carcinogenesis of lung cancer in non-smokers. *Thoracic Cancer*.

[47] Megaraj V., Zhou X., Xie F., Liu Z., Yang W., Ding X. (2014). Role of CYP2A13 in the bioactivation and lung tumorigenicity of the tobacco-specific lung procarcinogen 4-(methylnitrosamino)-1-(3-pyridyl)-1-butanone: in vivo studies using a CYP2A13-humanized mouse model. *Carcinogenesis*.

[48] Nakano M., Fukushima Y., Yokota S. (2015). CYP2A7 pseudogene transcript affects CYP2A6 expression in human liver by acting as a decoy for miR-126. *Drug Metabolism and Disposition*.

[49] Damkier P., Kjaersgaard A., Barker K. A. (2017). CYP2C19^∗^2 and CYP2C191^∗^7 variants and effect of tamoxifen on breast cancer recurrence: analysis of the International Tamoxifen Pharmacogenomics Consortium dataset. *Scientific Reports*.

[50] Justenhoven C., Pentimalli D., Rabstein S. (2014). Cyp2b66 is associated with increased breast cancer risk. *International Journal of Cancer*.

[51] Alunni-Fabbroni M., Ronsch K., Huber T. (2019). Circulating DNA as prognostic biomarker in patients with advanced hepatocellular carcinoma: a translational exploratory study from the soramic trial. *Journal of Translational Medicine*.

[52] Ashida R., Okamura Y., Ohshima K. (2018). The down-regulation of the CYP2C19 gene is associated with aggressive tumor potential and the poorer recurrence-free survival of hepatocellular carcinoma. *Oncotarget*.

[53] Nun-Anan P., Chonprasertsuk S., Siramolpiwat S. (2015). CYP2C19 genotype could be a predictive factor for aggressive manifestations of hepatocellular carcinoma related with chronic hepatitis B infection in Thailand. *Asian Pacific Journal of Cancer Prevention*.

[54] Yamamoto M., Watanabe M., Inoue N. (2020). Association of CD58 polymorphisms and its protein expression with the development and prognosis of autoimmune thyroid diseases. *Immunological Investigations*.

[55] Zhang Y., Liu Q., Yang S., Liao Q. (2021). CD58 immunobiology at a glance. *Frontiers in Immunology*.

[56] Frangieh C. J., Melms J. C., Thakore P. I. (2021). Multimodal pooled Perturb-CITE-seq screens in patient models define mechanisms of cancer immune evasion. *Nature Genetics*.

[57] Mayer B., Lorenz C., Babic R. (1995). Expression of leukocyte cell adhesion molecules on gastric carcinomas: possible involvement of LFA-3 expression in the development of distant metastases. *International Journal of Cancer*.

[58] Grote P., Boon R. A. (2018). LncRNAs coming of age. *Circulation Research*.

[59] Yang F., Jiang Y., Lv L. Z. (2017). Long non-coding RNA XLOC_010235 correlates with poor prognosis and promotes tumorigenesis of hepatocellular carcinoma. *European Review for Medical and Pharmacological Sciences*.

[60] Zhang Q., He Y., Luo N. (2019). Landscape and dynamics of single immune cells in hepatocellular carcinoma. *Cell*.

[61] Yao R. W., Wang Y., Chen L. L. (2019). Cellular functions of long noncoding RNAs. *Nature Cell Biology*.

[62] Qian X., Zhao J., Yeung P. Y., Zhang Q. C., Kwok C. K. (2019). Revealing lncRNA structures and interactions by sequencing-based approaches. *Trends in Biochemical Sciences*.

[63] Ramanathan M., Porter D. F., Khavari P. A. (2019). Methods to study RNA-protein interactions. *Nature Methods*.

