# Supplementary figures and images for: Identification of Key Functional Modules and Immunomodulatory Regulators of Hepatocellular Carcinoma
Source: J Immunol Res. 2021 Aug 13;2021:1801873. doi: 10.1155/2021/1801873 (PMC8378952; doi:10.1155/2021/1801873)

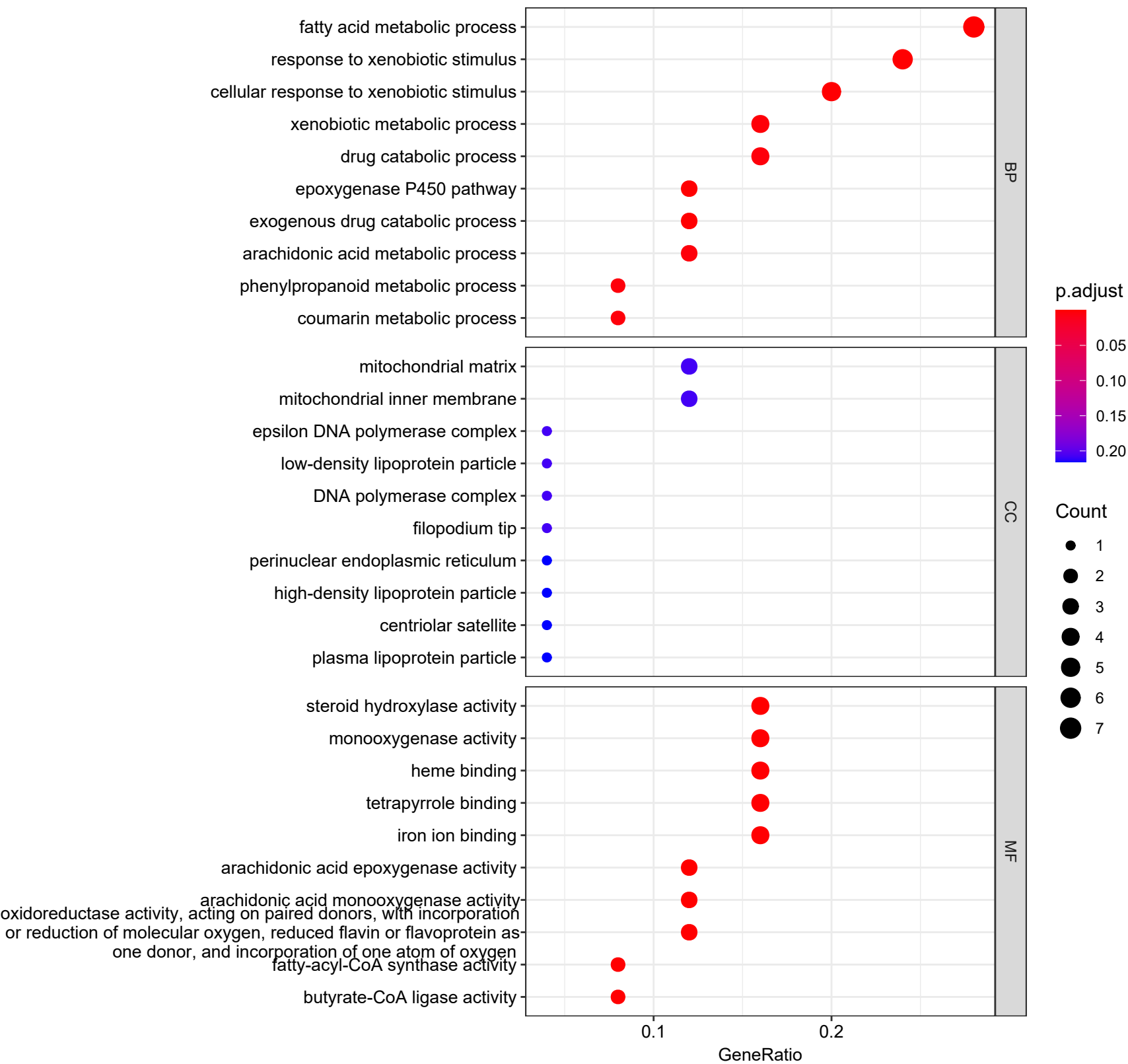

Supplement: Supplementary Materials — SFigure 1: enrichment analysis on the blue and yellow modules using (A) Gene Ontology and the (B) KEGG database. The significance of enrichment gradually increases from blue to red, and the dot size indicates the number of genes contained in the corresponding enriched term. [file 1801873.f1.zip › SFigure 1-A.pdf]

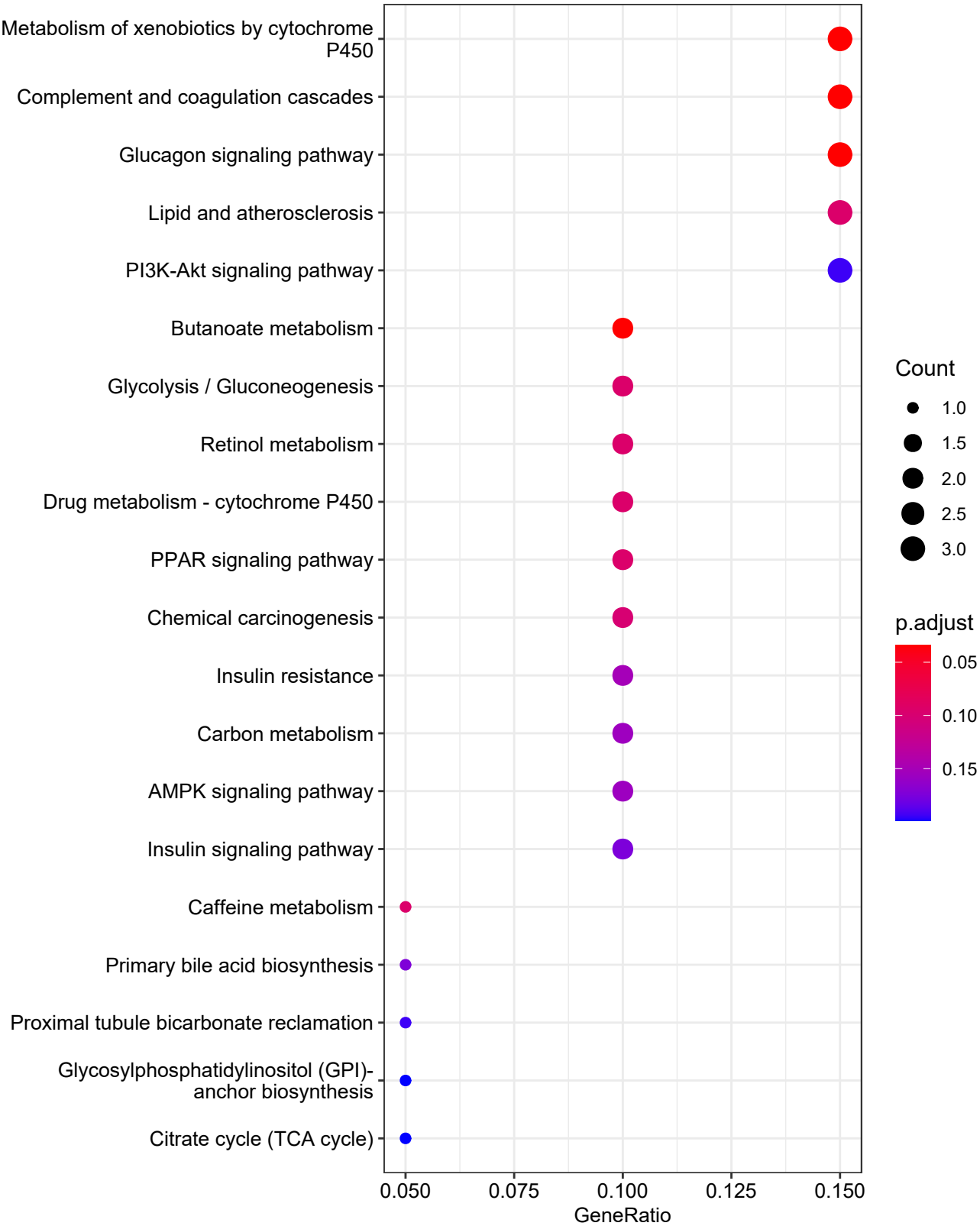

Supplement: Supplementary Materials — SFigure 1: enrichment analysis on the blue and yellow modules using (A) Gene Ontology and the (B) KEGG database. The significance of enrichment gradually increases from blue to red, and the dot size indicates the number of genes contained in the corresponding enriched term. [file 1801873.f1.zip › SFigure 1-B.pdf]
